# Guanxinjing capsule in the treatment of chronic stable angina: study protocol for a randomized controlled trial

**DOI:** 10.1186/s13063-018-2950-7

**Published:** 2018-10-20

**Authors:** Ying Tian, Junhua Zhang, Yingqiang Zhao, Jingyuan Mao, Linping Zhu, Rui Gao, Xuemei Wang, Mingjun Zhu, Lihong Ma, Mengyu Zhao, Wenke Zheng

**Affiliations:** 1 Tianjin University of Traditional Chinese Medicine, 312 Anshanxi Road, Nankai District, Tianjin, 300193 China; 2 The Second Hospital Affiliated to Tianjin University of Traditional Chinese Medicine, 69 Zengchan Road, Hebei District, Tianjin, 300010 China; 3 The First Hospital Affiliated to Tianjin University of Traditional Chinese Medicine, 314 Anshanxi Road, Nankai District, Tianjin, 300193 China; 4 Xiyuan Hospital of Chinese Academy of Traditional Chinese Medicine, Haidian District, Beijing, 100091 China; 5 Peking University First Hospital, 8 Xishiku Road, Xicheng District, Beijing, 1000343 China; 6 The First Hospital Affiliated to Henan University of Traditional Chinese Medicine, 19 Renmin Road, Zhengzhou City, 450000 Henan China; 7 Fuwai Hospital, National Center for Cardiovascular Diseases, 167 Beilishi Road, Xicheng District, Beijing, 100037 China

**Keywords:** Chinese herbal medicine formula, Angina pectoris, Qi deficiency and blood stasis syndrome, Guanxinjing capsule, Blinded, randomized controlled trial

## Abstract

**Background:**

Stable angina is a common cardiovascular disease with high mortality and a poor prognosis. Although there are various interventions against stable angina, none are able to significantly reduce the mortality rate. Guanxinjing capsule (GXJ) is made from the classical Chinese prescription Xuefuzhuyutang (血府逐瘀汤). Both basic research and clinical studies have shown that GXJ can relieve the symptoms of angina, but currently, the effects of GXJ lack high-quality clinical evidence. The aim of this study was to evaluate the clinical effectiveness and safety of GXJ compared with placebo.

**Methods/design:**

This multicentre, blinded, randomized trial will be conducted with a total of 120 participants diagnosed with chronic stable angina (Qi deficiency and blood stasis syndrome). Using a central randomization system, participants will be randomized (1:1) into groups receiving either GXJ or placebo for 8 weeks. After a 2-week run-in period, eligible patients will receive either GXJ or placebo (4 pills, three times daily) for 8 weeks in addition to conventional treatment. The primary outcomes include changes in the total exercise time on exercise tolerance tests and changes in the integral scores of angina symptoms. The secondary outcome measures include changes in the maximal estimated workload, changes in time to a 1 mm ST-segment depression or raise, changes in the time to onset of angina during exercise tolerance testing, changes in the total score of traditional Chinese medicine syndrome, and changes in the total score of the Generalized Anxiety Disorder 7-item assessment between baseline and week 8. Other outcome measures will also be assessed. All exercise tolerance tests use a standard Bruce multistage exercise test protocol. Adverse events will be monitored throughout the trial.

**Discussion:**

This study will investigate whether GXJ can alleviate clinical symptoms, increase the angina-free walking time, and improve quality of life in patients with chronic stable angina (Qi deficiency and blood stasis syndrome). The results of this study will provide clinical evidence for the application of GXJ to the treatment of stable angina.

**Trial registration:**

Chinese Clinical Trial Registry, ChiCTR1800014258. Registered on 2 January 2018.

**Electronic supplementary material:**

The online version of this article (10.1186/s13063-018-2950-7) contains supplementary material, which is available to authorized users.

## Background

Stable angina is a clinical syndrome characterized by discomfort in the chest, jaw, shoulder, back, or arms, typically elicited by exertion or emotional stress and relieved by rest or nitroglycerin [1]. Stable angina is a common and disabling disorder, and the prevalence is 3.6% in China [2]. Data from the literature report that the incidence of angina in the USA is 12.3 cases/1000 person years (age-adjusted, ages 45–74 years), with a prevalence of 20% in men older than 60 years, and similar numbers have been reported in Europe [3, 4]. The annual death rate of patients with stable angina is 1.2–2.4% [5]. This syndrome poses a large burden on the healthcare system and calls for continued research and the development of therapies, as well as prevention.

Chinese medicines have been used in therapeutic approaches in East Asia for more than two millennia. In Chinese medicine, angina pectoris is classified as “XiongBi” or “heartache” according to their symptoms, with a record dating from the Han dynasty and the accumulation of many effective treatments. In China, Chinese patent medicines are prevalent and are commonly used as an alternative to Western medicine to treat coronary heart disease (CHD) [6, 7]. Chinese medicines will be the optimal choice of complementary and alternative medicines for the treatment of angina pectoris.

From the perspective of traditional Chinese medicine (TCM), patients with CHD can be divided into different syndromes (Zheng), which is a summarization of the pathological changes that characterize the disease based on the location, the cause and nature of the disease, and the states of Xie (pathogenic factors) and Zheng (the healthy Qi). According to the data from a previous research regarding the syndrome differentiation of chronic stable angina, the syndrome of “Qi deficiency and blood stasis” might reach a proportion of 18.62% of clinical patients, which would rank first among the different subtypes [8]. A meta-analysis showed that reinforcing the Qi and activating the blood effectively ameliorates angina symptoms and demonstrates a safety profile among patients with CHD [9, 10].

In TCM theory, Qi represents a function. If the Qi is abundant, the function will be powerful, while if the Qi is insufficient, the function will be weak.

The Guanxinjing capsule (GXJ) was approved by the China Food and Drug Administration for the treatment of CHD in 2002. GXJ is based on a famous traditional Chinese prescription, Xuefuzhuyutang (血府逐瘀汤), which was created by Wang Qinren, a famous physician of the Qing dynasty, and has been widely used to treat various diseases caused by stasis. The GXJ capsule is composed of salvia, rhizomaligusticichuanxiong, red peony root, safflower, ginseng, polygonatum, *Panax notoginseng*, storax, and borneol, and functions to reinforce the Qi and activate the blood. Several studies have suggested that GXJ can increase myocardial hypoxia tolerance [11] and relieve the clinical symptoms of angina pectoris [12]. However, these studies were performed decades ago, with low-quality methods. In this study, we designed a prospective, randomized, multicentre, double-blind, placebo-controlled trial to investigate the effectiveness and safety of GXJ in adults with chronic stable angina, with the aim of providing powerful evidence for clinical application. This study is registered in the Chinese Clinical Trial Registry (ChiCTR1800014258).

In addition, depression and anxiety in patients with cardiac disease is highly prevalent and is independently associated with adverse cardiovascular consequences, including worse health-related quality of life, impaired functional status, recurrent cardiac events, and higher rates of mortality [13–17]. People with stable angina, an important subtype of cardiac disease, experience difficulties similar to those experienced by patients with other types of CHD, including depression, anxiety, and impaired quality of life [18–20]. Although studies found that antidepressant drug treatment did not improve the cardiovascular prognosis [21–23], we must consider the early psychological problems of patients with stable angina. Previous studies have suggested that GXJ can increase the concentration of 5-hydroxytryptamine (5-HT) and improve behavioural performance in depressed rats after heart attacks [24]. GXJ was recommended in the Consensus on the Diagnosis and Treatment of Depression before and after Percutaneous Coronary Intervention (PCI) [25] for the treatment of depression and/or anxiety before and after PCI. The Generalized Anxiety Disorder 7-item (GAD-7) test (see Additional file 1) is one of the few GAD measures that is also specifically linked to the Diagnostic and Statistical Manual of Mental Disorders, 4^th^ edition (DSM-IV) criteria. The GAD-7 test is a reliable tool for anxiety screening and diagnosis, as it uses patient self-evaluation, is easy to use, and has good reliability and validity in cardiovascular patients [26]. A score of 10 or higher on the GAD-7 test represents a reasonable cut point for identifying cases of GAD. Cut points of 5, 10, and 15 might be interpreted as representing mild, moderate, and severe levels of anxiety on the GAD-7 test. Construct validity was demonstrated by the fact that increasing scores on the GAD-7 scale were strongly associated with multiple domains of functional impairment [27]. In this study, we choose to use the GAD-7 scale as a screening tool to evaluate patient mental health to observe the effects of GXJ on patient mental states.

Based on the multifactorial effects of GXJ on CHD, our hypothesis is that GXJ, in addition to conventional treatments, can provide relief of angina symptoms in patients with chronic stable angina pectoris (Qi deficiency and blood stasis syndrome) when compared with conventional treatment alone. If successful, GXJ will represent a novel, promising alternative strategy for the further relief of angina symptoms.

### Objective

This study will evaluate the effectiveness and safety of the GXJ in adults with chronic stable angina pectoris (Qi deficiency and blood stasis syndrome) to clarify the role of GXJ in the medical field.

## Methods/design

### Study design

This study is a prospective, randomized, multicentre, blind, placebo-controlled trial.

The study is registered in the Chinese Clinical Trial Registry (ChiCTR1800014258) and complies with the principles of the Declaration of Helsinki and Good Clinical Practice (GCP) guidelines. Written informed consent will be obtained from all patients prior to their participation in this study, and the recruited patients will be randomly assigned to either the GXJ group or the placebo group. The protocol design is based on the following Standard Protocol Items: Recommendations for Interventional Trials (SPIRIT) checklist (see Additional file 2). We will rigorously follow the Consolidated Standards of Reporting Trials (CONSORT) Extension for Chinese Herbal Medicine Formulas 2017 recommendations in reporting the results [28].

**Fig. 1:**
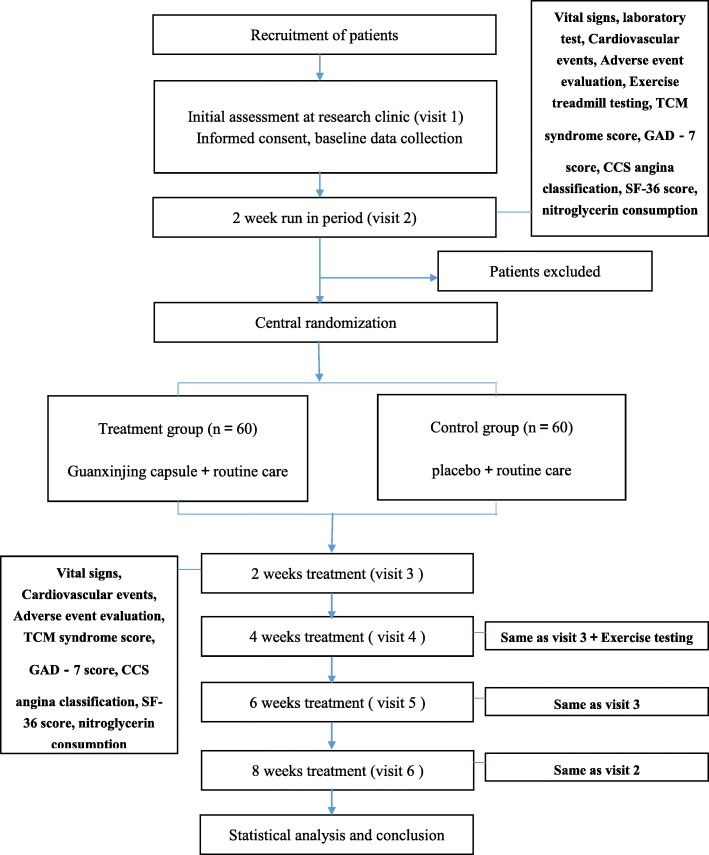
Flow diagram

The trial will be conducted at eight centres in China (see Additional file 3), and a total of 120 participants will be recruited. After the participants have provided consent, they will be enrolled in the trial, which consists of a 2-week run-in period and an 8-week treatment period. A flow diagram of the study procedures is illustrated in Fig. 1.

### Recruitment

Participants will be recruited through Internet advertisement and posters in the community and selected hospitals.

### Eligibility criteria

Eligible patients are those who meet all of the following inclusion criteria (listed in a subsequent section) and who do not have any of the listed exclusion criteria.

### Diagnostic criteria

The diagnostic criteria and definitions are listed as follows.

1. The diagnostic criteria for CHD are (1) a history of myocardial infarction, with or without revascularization (PCI or coronary artery bypass grafting) treatment; (2) coronary angiography or computed tomography coronary angiography confirmation of stenosis greater than 50% of at least one major branch of the coronary artery luminal diameter, with or without revascularization; or (3) noninvasive imaging stress test diagnostic of coronary artery disease (CAD) (e.g. nuclear perfusion scan, stress echocardiogram, or stress cardiac magnetic resonance scan). A patient meeting at least one of these criteria is considered to be diagnosed with CHD.
2. The diagnostic criteria for chronic stable angina were determined according to the guidelines for the diagnosis and treatment of chronic stable angina of China in 2007 [29]*,* the 2013 ESC guidelines on the management of stable coronary artery disease [30]*,* and the SIGN guideline on coronary revascularisation in the management of stable angina pectoris [31]. The classification of angina referred to the Canadian Cardiovascular Society (CSS) Functional Classification of Angina [32] (see Additional file 4).
3. The definition of major adverse cardiovascular event (MACE) is as follows: cardiac death (any death, unless an unequivocal noncardiac cause can be established), nonfatal myocardial infarction (MI) (appearance of pathological Q waves that were absent at baseline or a total creatine kinase [CK] level more than two times the upper limit of normal [ULN], with the presence of CK isoenzyme MB at greater levels than the ULN), or urgent revascularization with either PCI or coronary artery bypass graft.

### Standardization of traditional Chinese medicine differentiation

According to the technical guidelines for clinical research of TCM drugs and natural drugs treated for angina and coronary artery disease (2011) [33], the primary signs and symptoms are chest congestion and chest pain. Secondary signs and symptoms are palpitation, shortness of breath, fatigue, loss of energy, and a dark and purple complexion. In addition, the patient has a slight purple tongue and a weak and uneven pulse.

A patient with one of the primary symptoms and two of the secondary symptoms, combined with the tongue and pulse conditions, can be diagnosed as having Qi deficiency and blood stasis syndrome.

### Inclusion criteria

The inclusion criteria for the trial are as follows:

1. A diagnosis of CHD and the provision of exact imaging information (coronary angiography or computed tomography coronary angiography confirmation of stenosis greater than 50% of at least one major branch of the coronary artery luminal diameter, or nuclear perfusion scan diagnosed as CAD). CCS classification of angina grade II
2. TCM diagnosis of Qi deficiency and blood stasis syndrome, according to TCM standards
3. At least 2 months of standardized medication history before enrolment (sustaining the same medication, type, usage, and dose)
4. Age between 18 and 75, either male or female
5. Signed informed consent by participants or surrogates
6. Understanding and voluntary compliance with the research program, acceptance of the prescribed course and limits of the study, understanding the oral scale and diary card

### Exclusion criteria

The exclusion criteria for the trial are as follows:

1. Uncontrolled hypertension (systolic blood pressure ≥ 160 mmHg or diastolic blood pressure ≥ 100 mmHg). Severe cardiopulmonary insufficiency, or severe arrhythmia (rapid atrial fibrillation and flutter, paroxysmal ventricular tachycardia second degree and greater than a second degree atrioventricular [AV] block). Acute MI in the past 2 months, or has undergone coronary revascularization in the past 12 months
2. Renal insufficiency, male serum creatinine > 2.5 mg/ dl (> 220 μmol/L) or female serum creatinine > 2.0 mg/ dl (> 175 μmol/L). Serious liver disease (alanine transaminase and/or aspartate transaminase values two times higher than the upper reference limit value). Other severe primary disease, such as malignant tumour and haematopoietic system disease
3. Complications with absolute contraindications or relative contraindications during exercise tolerance testing (ETT) (e.g. severe aortic stenosis, acute aortic dissection, left primary coronary artery stenosis, acute myocarditis, or pericarditis)
4. Factors that precluded satisfactory interpretation of the electrocardiogram (ECG) (e.g. digoxin therapy, left bundle branch block, implanted with pacemaker, left ventricular hypertrophy, or electrolyte disturbance)
5. Complications with a serious bone joint disease or other comorbidities that may interfere with ability to perform required ETT
6. Patients planning to undergo coronary revascularization during the study period
7. Patients who might be allergic or are known to be allergic to ingredients of the study drug
8. Women who are pregnant or trying to become pregnant or are in the middle of a lactation period
9. Substance abuse, alcohol and drug dependence in the last 2 years
10. Other conditions that may affect the subjects’ adherence or lead to safety problems in the study
11. Participation in other clinical trials within the past 30 days

### Other criteria

#### Permitted and prohibited drugs

The following criteria apply to permitted and prohibited drugs:

1. Chinese herbs, drugs, and non-drug therapy which might alleviate angina or improve myocardial energy metabolism or upset restless symptoms are prohibited to use, including benzodiazepine sedatives and serotonin reuptake inhibitors.
2. Patients will be instructed to take a nitroglycerin tablet (0.5 mg per tablet) in case of angina pectoris, and this dose can be repeated approximately every 5 min until the angina is relieved. If the angina persists after three doses, the patient should be transported to a hospital immediately for further medical treatment. Patients will be asked to record the details of administration times, the number of doses, and the dosages of nitroglycerin in a patient diary, which will be collected by the investigators at a subsequent study visit.

Concomitant medications that are deemed necessary to manage any serious underlying diseases are allowed during the study. The names and amounts of concomitant medications should be recorded in detail in the patients’ medical records and case report forms (CRFs).

#### Suspension criteria

The criteria for suspension of participation are as follows:

1. Occurrence of serious adverse events (AEs), complications, or fatal physiological changes
2. Contravention of research program, such as the use of forbidden drugs prescribed by the program or not taking the medicine according to the research program (poor compliance)
3. Voluntary withdrawal
4. Withdrawal for various reasons, such as death or failure to attend follow-up visits
5. Incomplete data

### Interventions

#### Methods of administration

Eligible patients will be allocated to a treatment group or a control group. During the 2-week run-in period, all subjects will receive placebo capsules in addition to routine medications. Then, during the treatment period, eligible patients will be given GXJ capsules (4 pills, three times daily; China State Food and Drug Administration [SFDA] approval number Z20025812; composed of salvia, rhizomaligusticichuanxiong, red peony root, safflower, ginseng, polygonatum, *Panax notoginseng*, storax, and borneol) or placebo for 8 weeks, in addition to conventional treatment, which will maintain the same dosage. The GXJ and placebo capsules are provided by Baoding Traditional Chinese Medicine Pharmaceutical Co. Ltd, Anguo, China. Analyses have shown that the quality of the GXJ capsules is consistent with the Chinese Medicine Standards of the SFDA. The placebo capsule is similar to the GXJ capsule, with a comparable appearance. Each capsule is 0.3 g. The primary content of the placebo capsule is starch. By adding food colourants and flavouring agents, the mixture achieves a colour, smell, taste, and texture comparable to the contents of the GXJ capsule.

### Routine medications

Routine medications in this trial include antiplatelet medications, lipid-lowering medications, angiotensin-converting enzyme inhibitors or angiotensin receptor blockers, β-blockers, and calcium channel blockers, which must be maintained at the same dosage as prior to recruitment.

### Sample size

This is a clinical pilot trial; therefore, the total sample size of this study has been preliminarily determined to be 120 (during the trial, the expulsion rate is controlled within 20%) on expert advice. The treatment group and the control group will contain the same number of cases (that is, there will be 60 cases in each group).

### Randomization, allocation concealment mechanism, and blinding

Participants will be randomized in a 1:1 ratio, using a computer-generated, site-stratified, block randomization schedule. Randomization of the trial participants will be completed using an independent data centre (Tianjin Institute of Clinical Evaluation). A number will be placed in an envelope and subsequently sealed. The statistician will send the envelope directly to the product manufacturer for the labelling of the active and placebo products, according to the randomization number. The study capsules, labelled with sequential randomization numbers, will be sent to each research centre, and each patient will be assigned the lowest number available at each participating centre. All patients, care providers, and attending physicians will be blinded to the treatment assignments until the study is completed. Duplicated blinding codes will be provided to the primary research institution and the manufacturer to maintain, and the blinding codes cannot be broken until all the clinical data are entered into an EpiData database and locked, except in an emergency situation.

### Emergency unblinding

Only if a subject experiences an AE and the drug must be identified immediately can the unblinding letter be disassembled by the major investigator of the research unit. The reason for the unblinding will then be reported to the inspector. The subjects are withdrawn from the study after unblinding. Details of the unblinding cause, date, treatment situation, and results will be reported in the CRF and signed by the administrator.

### Content and points of data capture

The content and points of data capture in the trial are as follows:

1. Run-in period (14 days): 14 days before recruitment
2. Intervention period (8 weeks): follow-up every 2 weeks and recorded

Different items are measured according to the time points of data collection. The details are shown in Table 1.

### Outcome measurements

#### Primary outcomes

The primary outcomes of the study include change in total exercise time (time frame: baseline [visit 2] and week 8 [visit 6]) of ETT and the score of the angina symptom integral. Total exercise time was defined as the maximal duration of exercise that was performed by a patient in the ETT setting. All ETT tests used a standard Bruce multistage exercise test protocol. The angina symptom integral includes four contents: the severity of angina, the frequency of angina, the duration of angina, and the nitroglycerin dosage. The total score can range from 0 to 24, and higher scores indicate a worse status (see Table 1).

**Table 1:** Measurement items and points of data capture

Study phase	Run-in period	Intervention period				
	Visit 1	Visit 2	Visit 3	Visit 4	Visit 5	Visit 6
Time	-14 days	0 day	2 weeks	4 weeks	6 weeks	8 weeks
Baseline data collection						
Informed consent	×					
Inclusion/exclusion criteria	×					
Demographic data	×					
Get the central random number	×					
Concomitant disease and treatment	×					
Previous history、medical history and allergies	×					
Safety evaluation						
Vital signs	×	×	×	×	×	×
Complete blood count, urine and stool tests		×				×
Liver and renal function tests		×				×
Coagulation function test		×				×
Cardiovascular events	×	×	×	×	×	×
Adverse event evaluation	×	×	×	×	×	×
Efficiency evaluation						
Exercise treadmill testing’		×		×		×
TCM syndrome score	×	×	×	×	×	×
GAD-7 score	×	×	×	×	×	×
CCS angina classification	×	×	×	×	×	×
SF-36 score	×	×	×	×	×	×
Nitroglycerin consumption	×	×	×	×	×	×
Other work						
Concomitant medications	×	×	×	×	×	×
Dispense drug	×	×	×	×	×	
Recovery and record of study drug		×	×	×	×	×
Evaluate the adherence		×	×	×	×	×
Study completion status			×	×	×	×
CRF examination						×

*Abbreviations*: *SF36* 36-item Short Form Health Survey, *TCM* traditional Chinese medicine, *CCS* Canadian Cardiovascular Society, *GAD-7* Generalised Anxiety Disorder Assessment , *CRF* Case Report Form

#### Secondary outcomes

The secondary outcome measures include:

1. Changes in maximal estimated workload (in metabolic equivalents [METS]) during ETT between baseline and week 8
2. Changes in time to 1 mm ST-segment depression or raise during ETT at the end of the treatment period
3. Changes in time to onset of angina during ETT at the end of the treatment period
4. The total score of the TCM syndrome
5. The total score of the GAD-7 test

#### Other outcome measures

Other outcome measures include the following:

1. A 36-item Short Form Health Survey (SF-36) score
2. Consumption of short-acting nitrates
3. Angina grade, according to the CCS Angina Grading Scale
4. The probability of a MACE occurrence

#### Safety outcomes

Safety outcomes includes vital signs (temperature, heart rate, breathing, and blood pressure after 10 min of rest), a complete blood count, urine and stool tests, kidney and liver function tests, coagulation function test, and an evaluation of the occurrence of AEs.

### Adverse events

Every AE occurring during the study must be recorded in the CRF, according to the actual circumstances. The following information should be recorded: occurrence time, severity, duration, adopted measure, and outcome of the AE. The researcher should evaluate the correlation between the AE and the drug and decide whether to stop the observation, according to the condition, and follow-up investigations should be conducted on the cases that are discontinued due to adverse reactions.

### Quality control of the intervention

Quality control will be conducted by rigorous monitoring throughout the trial. All of the staff, including the operators, investigators, data collectors and analysers, are to be strictly trained to ensure that all practices at each hospital are generalized and standardized according to the standard operating procedures. Physicians must pass the required training test to demonstrate an understanding of the purpose and content of the trial, intervention strategies, and any AE observations. A multicentre test coordination committee, with a general manager of clinical research, will be established and will be responsible for the coordination of the clinical trials and for solving the relevant problems. The organizer will appoint monitors who will visit the clinical centres regularly to ensure that the records of the trial are complete and accurate, as well as to supervise the implementation of clinical trial programs.

The ETT method refers to the 2013 American Heart Association exercise standards for testing and training [34]. Eligible patients enter a single-blind, placebo-treatment, qualifying phase, during which they undergo Bruce [34] exercise treadmill tests. A standard 12-lead ECG with the patient in a supine position is obtained before each exercise treadmill test, and torso ECGs with the patient standing are monitored throughout the exercise testing. A core ECG laboratory (Tianjin University of TCM), blinded to treatment assignment, will interpret all rest and exercise ECGs. Exercise-induced ECG ischaemia is defined as the new development of horizontal or down-sloping ST-segment depression (1 mm at 80 ms after the J point) compared to baseline tracing. For patients with a permitted baseline ST-depression at rest (1 mm), qualifying ST-segment depression is defined as additional ST depression of at least 1 mm below the resting value. Subsequent exercise tests are performed approximately 2 h after morning dosing at 4 and 8 weeks after randomization.

At each visit, the investigators will record the number of pills the patients receive, use, and return, in detail, and record this information on the CRF without delay to evaluate the adherence of the subjects. Unused study drugs are counted. Drug compliance should be between 80 and 120%, with compliance = practical dose/application dose × 100%. The diary of angina episodes listed by the patient will be retrieved and maintained after the study. To maximize compliance and retention throughout the study, all participants will receive a telephone call before each follow-up appointment to remind them of the treatment time and location. Additionally, we will attempt to prevent dropouts by providing ongoing support to participants in the form of transport allowance, free medical examinations related to the research.

### Statistical analysis

Statistical analysis will be performed by the Tianjin Institute of Clinical Evaluation, and the data will be analysed using Statistical Analysis System (SAS) software version 9.3 (SAS Institute Inc., Cary, NC, USA). All effectiveness and safety analyses will be strictly conducted according to the intention-to-treat (ITT) principle, the full analysis set (FAS) population, the per protocol set (PPS) population, and the safety set (SS) population. Continuous variables will be described using means and standard deviations and tested with Student’s *t* tests. Categorical variables will be presented using percentages and tested with chi-squared tests. More details will be described in a formal statistical analysis plan.

### Data management

The patient’s information will be added to the paper CRF by researchers and reviewed by the clinical examiner. The first copy of the CRF will then be forwarded to the data administrator for data entry and management. To ensure the accuracy of the data, two data entry staff should independently input the data, and the data should be checked twice.

Data lockup will be implemented after the blinding state data review has confirmed the data to be correct. Researchers will be unable to modify data after locking.

### Ethical issues

#### Ethics statement

Researchers are responsible for ensuring that the study is conducted in accordance with the principles of the Declaration of Helsinki and GCP. Participants will voluntarily provide their written informed consent before any study procedures, and they can voluntarily withdraw from the study for any reason.

#### Ethical approval

This trial protocol was approved by the Ethics Committee of the Tianjin University of TCM (TJUTCM-EC20170006). The study protocol and informed consent procedure are consistent with scientific and ethical requirements. Written informed consent must be obtained from all participants or their legally authorized representatives before enrolment.

### Protocol amendments

If there are any problems during the implementation of the plan and it becomes necessary to revise it, the revised plan will be submitted to the ethics committee for approval before implementation.

## Discussion

Atherosclerotic cardiovascular disease (CVD) is a chronic disorder that develops insidiously throughout life and usually has progressed to an advanced stage by the time symptoms occur, such as angina. According to World Health Organization statistics, the annual global CVD mortality is predicted to be 23.6 million by 2030, and more people die annually from CVD than from any other cause, with an estimated 17.5 million deaths in 2012 (46% of all noninfectious chronic disease [NCD] deaths) [35]. Although a number of Western medications have been effective in reducing all-cause or CVD mortality, CVD remains a problem everywhere. Many patients do not receive conventional treatments due to side effects, contraindications, and drug-drug interactions [30], and many still experience angina and ischaemia at low workloads, despite undergoing standard treatments. Therefore, a broader range of therapeutic agents than are currently available is required to improve the quality of life for these patients.

TCM has been used for thousands of years to treat diseases. Many classic formulas have been modified by modern techniques and have been applied in clinical practice. Recently, there is a growing and sustained interest in the benefits of TCM and potential drug interactions with Western medications, especially for patients with stable angina. GXJ is a type of Chinese patent medicine that is used to treat Qi deficiency and blood stasis syndromes. The primary ingredients are salvia (Danshen), rhizomaligusticichuanxiong (Chuanxiong), red peony root (Chishao), safflower (Honghua), ginseng (Renshen), polygonatum (Yuzhu), *Panax notoginseng* (Sanqi), storax (Suhexiang), and borneol (bingpia), with the functions of reinforcing the Qi and activating the blood. Qi deficiency and blood stasis provide the crucial pathogenesis of stable angina in the TCM concept. We designed this trial with the hope of verifying that GXJ plus conventional treatment can provide relief of angina symptom in patients with chronic stable angina pectoris (Qi deficiency and blood stasis syndrome). If successful, this treatment will represent a novel, promising, alternative strategy for further relieving angina symptoms.

The study design has two primary points. (1) This is a superiority trial comparing a study drug with placebo, selecting chronic stable angina patients with exact imaging information. (2) By adopting the clinical effect evaluation methods of “the combination of disease and syndrome, multidimension index” [36], we selected indices that reflect both the advantages and characteristics of TCM and the conventional efficacy evaluation of Western medicine, setting the changes in total exercise time during ETT tests and the changes in the integral score of angina symptoms as the primary outcomes and the total score of TCM syndrome as the secondary outcome measure. We will also observe the changes in the total scores of the GAD-7 test to provide a direction for further clinical trials. This protocol has been developed according to the SPIRIT 2013 statement and the SPIRIT 2013 explanation and elaboration: guidance for protocols of clinical trials to establish appropriate standards for the scientific, ethical, and safety issues of the study.

However, there are several limitations in this study. One of the major drawbacks is the lack of assessment of the long-term effects of GXJ on primary outcome measures. In our study, the treatment period is only 56 days, which is relatively short. Because of the short length of the follow-up period, the potential roles of GXJ in reducing major vascular events and overall mortality over the long term will remain unknown. Therefore, well-designed randomized controlled trials that compare GXJ with conventional anti-anginal therapies and using longer follow-up periods are required in the future.

### Trial status

Currently, participant recruitment is ongoing.

## Additional files

Additional file 1: GAD-7 (General Anxiety Disorder-7) test. (DOCX 50 kb)

Additional file 2: SPIRIT checklist. (DOC 121 kb)

Additional file 3: Research settings. (DOCX 56 kb)

Additional file 4: CSS Functional Classification of Angina. (DOCX 55 kb)

Additional file 5: Model consent form [in Chinese]. (DOCX 20 kb)

## Abbreviations

AE: Adverse event
CAD: Coronary artery disease
CCS: Canadian Cardiovascular Society
CHD: Coronary heart disease
CRF: Case report form
CVD: Cardiovascular disease
ECG: Electrocardiogram
ETT: Exercise tolerance testing
GAD-7: Generalized Anxiety Disorder 7-item (assessment)
MACE: Major adverse cardiovascular event
MET: Metabolic equivalent
NCD: Noninfectious chronic disease
PCI: Percutaneous coronary intervention
SF-36: 36-item Short Form Health Survey
TCM: Traditional Chinese medicine
