# Supplementary material for: Guanxinjing capsule in the treatment of chronic stable angina: study protocol for a randomized controlled trial
Source: Trials. 2018 Oct 20;19:577. doi: 10.1186/s13063-018-2950-7 (PMC6196008; doi:10.1186/s13063-018-2950-7)
Supplement: Supplementary file 1 — GAD-7 (General Anxiety Disorder-7) test. (DOCX 50 kb) [file 13063_2018_2950_MOESM1_ESM.docx]

# Additional file 1：

# GAD-7 (General Anxiety Disorder-7)

| Over the last 2 weeks, how often have you been bothered by the following problems? | Not at all | Several days | More than half the days | Nearly every  day |
| --- | --- | --- | --- | --- |
| 1. Feeling nervous, anxious or on edge | 0 | 1 | 2 | 3 |
| 2. Not being able to stop or control worrying | 0 | 1 | 2 | 3 |
| 3. Worrying too much about different things | 0 | 1 | 2 | 3 |
| 4. Trouble relaxing | 0 | 1 | 2 | 3 |
| 5. Being so restless that it is hard to sit still | 0 | 1 | 2 | 3 |
| 6. Becoming easily annoyed or irritable | 0 | 1 | 2 | 3 |
| 7. Feeling afraid as if something awful might happen | 0 | 1 | 2 | 3 |
| Total score =add columns + + | | | | |
