# Supplementary material for: Guanxinjing capsule in the treatment of chronic stable angina: study protocol for a randomized controlled trial
Source: Trials. 2018 Oct 20;19:577. doi: 10.1186/s13063-018-2950-7 (PMC6196008; doi:10.1186/s13063-018-2950-7)
Supplement: Supplementary file 3 — Research settings. (DOCX 56 kb) [file 13063_2018_2950_MOESM3_ESM.docx]

# Additional file 3：

**Research settings**

| **Research setting** |
| --- |
| **The First Hospital Affiliated to Tianjin University of Traditional Chinese Medicine** |
| **The Second Hospital Affiliated to Tianjin University of Traditional Chinese Medicine** |
| **The First Hospital Affiliated to Henan University of Traditional Chinese Medicine** |
| **Xiyuan Hospital of Chinese Academy of Traditional Chinese Medicine** |
| **Peking University First Hospital** |
| **Fuwai Cardiovascular Disease Hospital of Chinese Academy of Medical Sciences** |
| **Second Hospital of Hebei Medical University** |
| **Traditional Chinese Medicine Hospital of Shanxi Provincial** |
