# Supplementary material for: Guanxinjing capsule in the treatment of chronic stable angina: study protocol for a randomized controlled trial
Source: Trials. 2018 Oct 20;19:577. doi: 10.1186/s13063-018-2950-7 (PMC6196008; doi:10.1186/s13063-018-2950-7)
Supplement: Supplementary file 4 — CSS Functional Classification of Angina. (DOCX 55 kb) [file 13063_2018_2950_MOESM4_ESM.docx]

| Class I | Ordinary activity does not cause angina such as walking and climbing stairs. Angina with strenuous or rapid or prolonged exertion at work or recreation. |
| --- | --- |
| Class II | Slight limitation of ordinary activity. Angina on walking or climbing stairs rapidly, walking or stair climbing after meals, or in cold, wind or under emotional stress, or only during the first few hours after awakening. Walking more than two blocks on the level and climbing more than one flight of ordinary stairs at a normal pace and in normal conditions. |
| Class III | Marked limitation of ordinary physical activity. Angina on walking one to two blocksa on the level or one flight of stairs in normal conditions and at a normal pace. |
| Class IV | Inability to carry on any physical activity without discomfort' – angina syndrome may be present at rest |

# Additional file 4：

Canadian Cardiovascular Society (CSS) Functional Classification of Angina
