# Supplementary material for: Guanxinjing capsule in the treatment of chronic stable angina: study protocol for a randomized controlled trial
Source: Trials. 2018 Oct 20;19:577. doi: 10.1186/s13063-018-2950-7 (PMC6196008; doi:10.1186/s13063-018-2950-7)
Supplement: Supplementary file 5 — Model consent form [in Chinese]. (DOCX 20 kb) [file 13063_2018_2950_MOESM5_ESM.docx]

**受试者须知**

**尊敬的 先生/女士**

您被邀请参加由天津中医药大学第二附属医院心内科负责组织开展的， 医院参加的“冠心静胶囊治疗慢性稳定性心绞痛（气虚血瘀证）临床试验”项目。您在决定是否参加这个项目之前，请仔细阅读以下内容，它将帮助您了解这项研究的价值与意义，以及参加这项研究可能给您带来的益处与风险。

**一、研究的背景和目的**

1、研究背景

冠心病即冠状动脉性心脏病(Coronary Heart Disease， CHD)，是指由于冠状动脉粥样硬化或血栓形成、冠状动脉痉挛等原因引起冠状动脉官腔狭窄或阻塞，从而导致心肌缺血缺氧，甚至局部坏死的心脏病。本病是一种常见的心血管疾病，具有高发病率、高致死率及高致残率的特点，严重威胁人类健康。随着社会－心理－生物医学模式兴起，冠心病与患者心理不良情绪变化之间的关系越来越受到关注，2012年《欧洲心血管疾病预防临床实践指南》明确指出焦虑、抑郁等心理社会因素是心血管疾病发生的危险因素并对疾病的发展和预后有不良影响。目前已有大量的研究围绕冠心病合并焦虑／抑郁障碍展开，然而当前的药物治疗方案在花费大量医疗支出的同时仅能轻度改善患者的焦虑抑郁情绪，未能改善心血管病预后，研究结果并不理想，这提示我们应当将关注的重心前移，对于焦虑／抑郁障碍诊断之前的心绪异常加以干预，从而延缓冠心病患者中焦虑／抑郁的发生，提高患者的生活质量。

冠心病是一种心身疾病，其发生发展与行为特征和情绪应激有着密切的关系。中医学认为，冠心病的产生，是心之气血失衡造成的，除表现为胸闷气短、痛有定处、心胸隐痛、动则益甚、心悸等外，还可表现在气血失衡导致的心神失养，可表现为神志改变，如心烦不宁、情志抑郁、失眠多梦、健忘等症状。从气-血-神相关理论论治冠心病，可从根本上把握其基本病机，以益气活血，安神定志作为冠心病的治疗法则，通过调和气血达到调整脏腑功能、形神同治的目的。

冠心静胶囊是保定中药制药股份有限公司的独家产品(国药准字Z20025812)，由丹参、赤芍、川芎、红花、玉竹、三七、人参、苏合香、冰片组成，具有活血化瘀、益气通脉的功效，用于气虚血瘀引起的胸痹胸痛，气短心悸及冠心病见上述症状者。

该药以清代王清任《医林改错》血府逐瘀汤为基础，根据中医药理论加减方中诸药，经工艺技术改进研制而成，于1993年由河北省卫生厅批准上市，2002年上升为国家药品标准，2012年标准转正。

冠心静胶囊在血府逐瘀汤基础上进行精简调整，增加益气养阴之人参、玉竹，并以冰片、苏合香之辛香窜达取代柴胡、枳壳等理气之品，增加行气止痛之力，并能引药入神窍，增加对中枢神经系统的影响，使组方既有改善心肌缺血症状、调节血脂、有效控制动脉粥样硬化的进程等有利于心血管疾病治疗的作用，又能调适患者的心理情智，为冠心病合并情绪异常患者提供了治疗选择。

既往冠心静胶囊临床研究较少，对其临床疗效及安全性仍需进一步加强研究。因此，需要组织开展多中心的随机对照研究，进一步评价冠心静胶囊治疗慢性稳定性心绞痛的疗效和安全性。

2.研究目的

评价冠心静胶囊治疗慢性稳定性心绞痛（气虚血瘀证）的疗效和安全性，并探索其对冠心病患者情绪方面的影响。

**二、研究过程**

本研究为随机对照多中心临床试验，全国将有120例符合条件的慢性稳定性心绞痛患者参加，本中心计划邀请20位患者参加该项目。如果您同意参加此项目，并符合参加的条件，您将在自愿签署知情同意书后进入临床试验。本项目分为三阶段实施：

**第一阶段：筛选期**

医生将了解您的病史，进行相应的医学检查和化验。如果您的检查结果不符合进入第二阶段的要求，您将不再参加第二阶段的研究。

**第二阶段：导入期**

在通过了第一阶段的筛选后，您将进入为期2周的导入期。导入期间，您只需按照日常用药即可。导入期结束时，您需要在您的医生指导下参加一次平板试验，以评估您的心功能情况。

**第三阶段：治疗期**

在通过了第二阶段的导入期后，您将进入为期8周的慢性稳定性心绞痛治疗期。本研究采用随机入组的方式，您既有可能入选试验组，也有可能入选对照组。您入选不同的组别不会影响医生对您的常规治疗。

试验组是在日常基础用药的基础上再服用冠心静胶囊，每天三次，每次4粒，连续服用8周。对照组是在日常基础用药基础上，服用冠心静胶囊模拟剂，每天三次，每次4粒，连续服用8周。在治疗期内，医生会详细询问并记录您的病情变化，进行与本项目相关的医学项目检查，包括量表、仪器和化验检查。

本项目所使用的试验药物可能会对您的病情具有改善做用，因个体差异不同，改善的程度亦可能有所不同。参加本项研究需要您遵循以下要求：

1. 在治疗期内，切勿自行使用本项研究中不允许联合应用的治疗药物。
2. 严格按照医生的要求使用药物和接受相关检查。

**三：受试者权益**

本研究将记录您的个人情况和与疾病相关的资料，包括：年龄、性别、身高、体重、民族、职业等；病史（心肌梗死、血运重建术等）、过敏史、病程、合并疾病及用药情况等；生命体征(静息10分钟的血压、体温、呼吸、心率)。

在研究过程中，您将免费使用试验药物，如治疗期间，所有相关的检查项目均也是免费的。医生会将这些检测结果告知您，这将有助于您了解自身疾病的变化，也为进一步诊治您的疾病提供帮助。您在治疗期间的其他常规治疗和检查措施均正常收费。在研究过程中，如果出现以下情况，我们将及时告知您，并终止您继续参加本项目：

1. 用药过程中发生严重安全性问题；
2. 研究中发现药物治疗效果太差，甚至无效，不具有临床价值；
3. 在研究中发现临床研究方案有重大失误，难以评价药物效应；或者在实施中发生了重要偏差，再继续下去，难以评价药物效应。
4. 药政主管部门或申办者决定终止本项研究。

四、受试者隐私保护

本项研究所需要的各种资料记录中，您的姓名会被拼音缩写代替。本项研究的结果可能会在医学专业杂志上发表，但您的所有个人信息将被严格保密。医学伦理委员会和相关研究人员在必要时，经过批准可以查阅您的医学资料，但其他人不会获得您的个人资料。

在参加本项研究的全过程中，您都是自愿的。您可以不同意参加本研究，或者已经参加了本研究也可以再任何时间、无需任何理由的退出。您的这些决定均不会影响对您的正常治疗。如果您同意参加，您本人或您的代理人需要在知情同意书上签字。

五、在研究中可能的受益及承受的风险和不适，以及研究者拟采取的风险防范措施

研究药物冠心静胶囊上市至今，未检索到不良反应相关文献，2014年，国家药品不良反应监测系统病例报告数据库（河北省）检索到其不良反应为胸闷、呼吸困难、烧心、反酸、胃酸过多、恶心、腹痛、腹泻、便秘、失眠，头痛。

如果出现上述可预见的药物不良反应，或尚未发现的、不可预见的药物不良反应，医生会根据您的具体情况采取及时的处理措施，以降低可能发生的风险。如果您在本项研究过程中发生了严重不良反应，您将得到积极治疗。如果确认您所出现的不良事件/反应与研究药物冠心静胶囊有关，依据国家法律法规及药物临床试验管理规范的要求，有保定中药制药有限公司承担与不良反应相关的治疗费用和相应的赔偿。

遇到紧急情况请联系研究者： 联系电话：

如果得到可能影响受试者继续参加本项目的信息，受试者或其合法代理人将及时得到通报。

**如果您已经全部理解了以上内容，并同意参加此项临床试验，请签署知情同意书。**

**受试者知情同意书**

项目名称：冠心静胶囊治疗慢性稳定性心绞痛（气虚血瘀证）临床试验

本项目已经获得本项研究组长单位天津中医药大学第二附属医院医学伦理委员会和您所在医院医学伦理委员会的批准。

天津中医药大学第二附属医院医学伦理委员会联系电话：

您所在医院医学伦理委员会联系电话：

负责该项研究的医生已向我解释说明了临床研究的内容，我有机会提问，并且我的所有疑问已经获得解答，而且我已经全部理解了这些解答。

我自愿参加本项研究。我可以随时退出本项研究，而且这样做不会妨碍我在此医院应该得到的诊治权利。我有权获得一份知情同意书副本。

我已经认真阅读并完全理解受试者须知的内容，同意参加本项研究。

受试者签字： 联系电话： 签字日期：

（或代理人签字）： 联系电话： 签字日期：

研究者签字： 联系电话： 签字日期：
